# Interim Safety Profile From the Feasibility Study of the BrainGate Neural Interface System

**DOI:** 10.1212/WNL.0000000000201707

**Published:** 2023-03-14

**Authors:** Daniel B. Rubin, A. Bolu Ajiboye, Laurie Barefoot, Marguerite Bowker, Sydney S. Cash, David Chen, John P. Donoghue, Emad N. Eskandar, Gerhard Friehs, Carol Grant, Jaimie M. Henderson, Robert F. Kirsch, Rose Marujo, Maryam Masood, Stephen T. Mernoff, Jonathan P. Miller, Jon A. Mukand, Richard D. Penn, Jeremy Shefner, Krishna V. Shenoy, John D. Simeral, Jennifer A. Sweet, Benjamin L. Walter, Ziv M. Williams, Leigh R. Hochberg

**Affiliations:** From the Center for Neurotechnology and Neurorecovery (CNTR) (D.B.R., L.B., S.S.C., C.G., R.M., M.M., L.R.H.), Department of Neurology, and Department of Neurosurgery (Z.M.W.), Massachusetts General Hospital, Boston; Harvard Medical School (D.B.R., S.S.C., L.R.H.), Boston, MA; Department of Biomedical Engineering (A.B.A., R.F.K.), Case Western Reserve University, Cleveland, OH; FES Center of Excellence, Rehab. R&D Service (A.B.A., R.F.K., J.P.M., J.A.S., B.L.W.), Louis Stokes Cleveland Department of Veterans Affairs Medical Center, OH; Center for Neurorestoration and Neurotechnology (CfNN) (M.B., J.P.D., J.D.S., L.R.H.), Rehabilitation R&D Service, Department of Veterans Affairs Medical Center, Providence, RI; Legs and Walking Lab (D.C.), Shirley Ryan AbilityLab, Chicago, IL; Department of Physical Medicine and Rehabilitation (D.C.), Northwestern University Feinberg School of Medicine, Rehabilitation Institute of Chicago, IL; Department of Neuroscience (J.P.D.), Robert J. and Nancy D. Carney Institute for Brain Science (J.P.D., J.D.S., L.R.H.), School of Engineering (J.P.D., J.D.S., L.R.H.), and Department of Rehabilitation Medicine (J.A.M.), Brown University, Providence, RI; Department of Neurological Surgery (E.N.E.), Montefiore Medical Center, Albert Einstein College of Medicine, Bronx, NY; European University of Cyprus (G.F.), Nicosia, Cyprus; Department of Neurosurgery (J.M.H.), Stanford University School of Medicine, CA; Wu Tsai Neurosciences Institute (J.M.H., K.V.S.), Bio-X Institute (J.M.H., K.V.S.), and Departments of Neurobiology (K.V.S.), Electrical Engineering (K.V.S.), and Bioengineering (K.V.S.), Stanford University, CA; Department of Neurological Surgery (R.F.K., J.P.M., J.A.S.), University Hospitals Case Medical Center, Cleveland, OH; Neurology Section (S.T.M.), VA Providence Health Care System, Providence, RI; Department of Neurology (S.T.M.), Alpert Medical School of Brown University, Providence, RI; Sargent Rehabilitation Center (J.A.M.), Warwick, RI; Section of Neurosurgery (R.D.P.), Department of Surgery, University of Chicago; Department of Neurosurgery (R.D.P.), Rush University Medical Center, Chicago, IL; Department of Neurology (J.S.), Barrow Neurological Institute, Phoenix, AZ; Howard Hughes Medical Institute at Stanford University (K.V.S.); Center for Neurological Restoration (B.L.W.), Cleveland Clinic, OH; and Program in Neuroscience (Z.M.W.), Harvard-MIT Program in Health Sciences and Technology, Harvard Medical School, Boston, MA.

## Abstract

**Background and Objectives:**

Brain-computer interfaces (BCIs) are being developed to restore mobility, communication, and functional independence to people with paralysis. Though supported by decades of preclinical data, the safety of chronically implanted microelectrode array BCIs in humans is unknown. We report safety results from the prospective, open-label, nonrandomized BrainGate feasibility study (NCT00912041), the largest and longest-running clinical trial of an implanted BCI.

**Methods:**

Adults aged 18–75 years with quadriparesis from spinal cord injury, brainstem stroke, or motor neuron disease were enrolled through 7 clinical sites in the United States. Participants underwent surgical implantation of 1 or 2 microelectrode arrays in the motor cortex of the dominant cerebral hemisphere. The primary safety outcome was device-related serious adverse events (SAEs) requiring device explantation or resulting in death or permanently increased disability during the 1-year postimplant evaluation period. The secondary outcomes included the type and frequency of other adverse events and the feasibility of the BrainGate system for controlling a computer or other assistive technologies.

**Results:**

From 2004 to 2021, 14 adults enrolled in the BrainGate trial had devices surgically implanted. The average duration of device implantation was 872 days, yielding 12,203 days of safety experience. There were 68 device-related adverse events, including 6 device-related SAEs. The most common device-related adverse event was skin irritation around the percutaneous pedestal. There were no safety events that required device explantation, no unanticipated adverse device events, no intracranial infections, and no participant deaths or adverse events resulting in permanently increased disability related to the investigational device.

**Discussion:**

The BrainGate Neural Interface system has a safety record comparable with other chronically implanted medical devices. Given rapid recent advances in this technology and continued performance gains, these data suggest a favorable risk/benefit ratio in appropriately selected individuals to support ongoing research and development.

**Trial Registration Information:**

ClinicalTrials.gov Identifier: NCT00912041.

**Classification of Evidence:**

This study provides Class IV evidence that the neurosurgically placed BrainGate Neural Interface system is associated with a low rate of SAEs defined as those requiring device explantation, resulting in death, or resulting in permanently increased disability during the 1-year postimplant period.

For many people with paralysis, the cortical substrates of motor activity, speech, and cognition are intact but functionally disconnected from the nerves and muscles that enable movement and communication. Among the most physically disabled are individuals with locked-in syndrome, who have limited or no volitional muscle control.^1^ Cervical spinal cord injury (SCI), brainstem stroke, muscular dystrophy, or motor neuron disease can cause similar impairments in communication and functional independence. Brain-computer interfaces (BCIs) bypass the site of pathology, transmitting information directly from cerebral cortex to an assistive technology to restore communication and improve independence.^2^

There are several classes of BCIs under development that use different sensors and decoding algorithms.^3^ Intracortical BCIs use sensors that are surgically implanted on the cortical surface and can access information-rich single neurons and local field potentials without degradation in signal content from spatial averaging and bone filtering seen with nonsurgical approaches.^4^ Any implanted medical device must have an adequate safety profile before clinical use so that patients, caregivers, and healthcare professionals can make informed decisions about risks and benefits.

Two reviews have addressed the safety of implanted BCIs.^5,6^ One is limited to endovascular stent-electrode arrays and reports primarily on electrode performance and structural characteristics of the implanted venous sinus, which are not directly related to this study.^5^ The other review includes an analysis of intracortical microelectrode arrays (including some data inferred from BrainGate trial participants); however, the only safety metric reported was duration of device implantation, which was used as a surrogate marker of days without major complication.^6^

In 2004, the first trial of the BrainGate Neural Interface System was launched.^7^ The objectives of the ongoing prospective, open-label, nonrandomized feasibility trial are to assess the safety of the BrainGate system and its feasibility to control assistive technology by people with paralysis. The BrainGate trial has accrued more than 12,000 participant-days of safety data, with more than 17,000 array-days (some participants received 2 arrays simultaneously), including 2 research participants who used the system for more than 5 years.^8,9^ In this study, we report all safety data from all BrainGate clinical trials from 2004 to December 31, 2021. We compared BrainGate's safety profile with other implanted devices approved for neurologic disorders including epilepsy and movement disorders and seek to answer the primary research question of whether the neurosurgically placed BrainGate Neural Interface System was associated with a low rate of serious adverse events (SAEs) defined as those requiring device explantation, resulting in death, or resulting in permanently increased disability during the 1-year postimplant period.

## Methods

The BrainGate consortium is engaged in a multicenter, prospective, open-label, nonrandomized feasibility study (ClinicalTrials.gov Identifier: NCT00912041) of an implanted BCI system (Figure). The study is performed under an Investigational Device Exemption (IDE) from the US Food and Drug Administration and approved by the Mass General Brigham Institutional Review Board (Protocol #: 2009P000505; CAUTION: Investigational Device. Limited by Federal Law to Investigational Use). Adults aged 18–75 years with quadriparesis caused by SCI, brainstem stroke, motor neuron disease, or muscular dystrophy have been recruited primarily through referral by a neurologist or other clinician at 1 of 7 clinical sites in the United States (Tables 1 and 2). Please see the eMethods, links.lww.com/WNL/C565 section in the Supplement for additional details.

**Figure:**
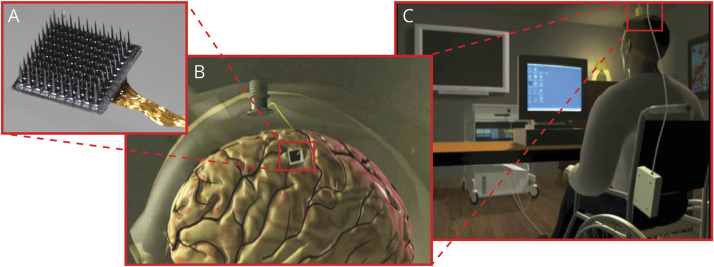
Components of the Investigational BrainGate Neural Interface System (A) The recording sensor is a “Utah” microelectrode array (NeuroPort, prior manufacturer: Cyberkinetics Neurotechnology Systems; present manufacturer, Blackrock Neurotech; both, Salt Lake City, UT) consisting of 96 electrodes arranged on a 4 × 4-mm platform. During surgical placement, the microelectrode array is placed onto the cortical surface of the predetermined brain region of interest and a pneumatic inserter wand is used to apply a precise amount of force to the array, inserting the microelectrodes through the pial surface and into the upper layers of cortex. (B) The microelectrode array is connected through a bundle of gold wires to a percutaneous pedestal, which is affixed to the outer table of the cranium by titanium surgical screws. When not in use, the percutaneous pedestal is covered by a disposable fitted cap. (C) Signals from the pedestal are transmitted either wirelessly^10^ or through a cable to a signal processor, decoding computers, and output devices. Images published with permission from the authors.

**Table 1:**
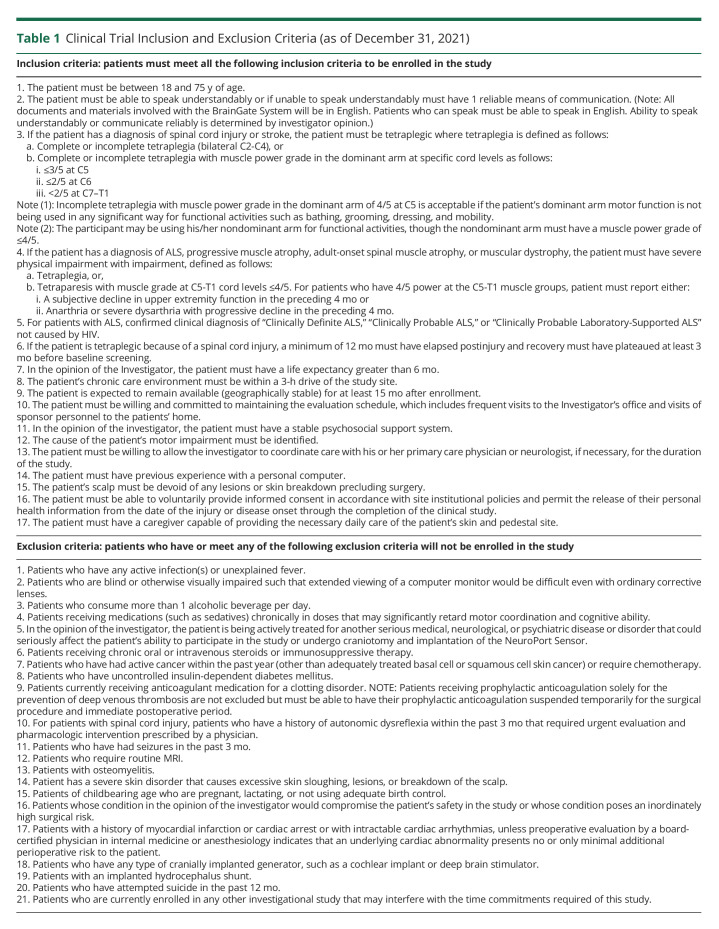
Clinical Trial Inclusion and Exclusion Criteria (as of December 31, 2021)

**Table 2:**
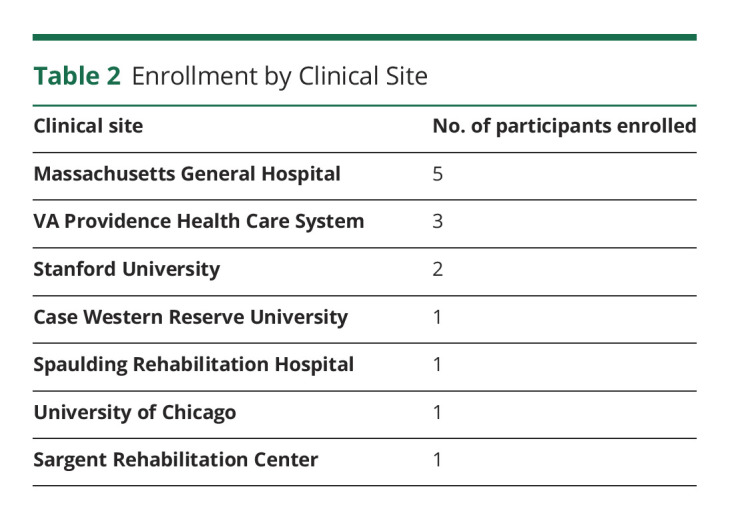
Enrollment by Clinical Site

## Results

Between 2004 and December 2021, 14 people enrolled in the BrainGate trial (Tables 2 and 3), of whom 2 are currently engaged in research. Four people were enrolled in a first-generation BrainGate clinical trial between 2004 and 2009. One participant was enrolled in a first-generation BrainGate trial when it ended in 2009; she transitioned into and enrolled in the second-generation BrainGate trial and participated for another 2 years. In addition to that “transitional” participant, 10 people, including 2 active participants, enrolled in the second-generation BrainGate trial.

**Table 3:**
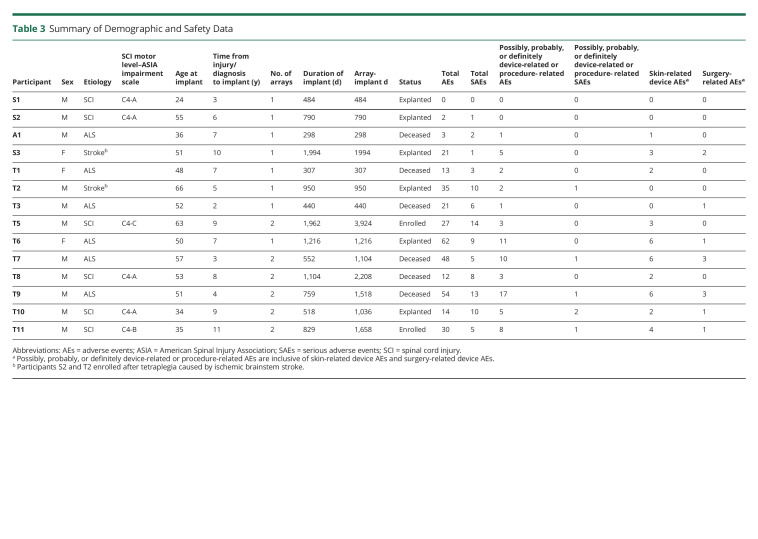
Summary of Demographic and Safety Data

Three participants were female and 11 were male. Eleven participants identified as White, 1 as Asian, 1 as Black, and 1 as Other. One participant identified as Hispanic ethnicity. All participants had quadriparesis, including 6 with SCI, 6 with ALS, and 2 with brainstem stroke. The median age at array implantation was 51 years (range 24–66). For participants with SCI or brainstem stroke, the median time from injury or stroke to implant was 8.5 years (range 3–11). For participants with ALS, the median time from diagnosis to implant was 5.5 years (range 2–7); we note that people with ALS often have symptoms for months or years before receiving a formal diagnosis. The median duration of device implantation was 774 days (range 296–1,994). There were 12,203 implant-days across all 14 participants (or 17,927 array-implant-days, counting participants with 2 arrays twice). Six participants underwent elective explant of the BrainGate device at the conclusion of their participation in the trial; 1 participant requested removal of the percutaneous pedestal but not the intracranial microelectrode array. Six participants died of progression of their underlying neurologic disease while enrolled. In no case was a participant’s death related to the investigational device or clinical trial; there were no unanticipated adverse device effects. Other than transitioning in 2013 from placing 1 to 2 arrays per participant and the introduction of a wireless signal transmitter in 2017,^10^ there have been no substantial modifications to the underlying hardware.

Among the 14 participants, there were 342 adverse events (AEs); the median number of AEs per participant was 21, and the range was from 0–62 AEs (Table 3). Two hundred seventy-four of the 342 (80.1%) AEs were unrelated to the investigational device; most were medical events common in people with tetraplegia (e.g., urinary tract infection, pneumonia, and decubitus ulcer). There were no device-related AEs that resulted in a participant's death, a participant exiting the clinical trial, a need to explant the device, or the inability to continue participating in research sessions.

Of the 68 device-related AEs (median 2 per participant, range 0–17), approximately half (35/68) were related to irritation, sensitivity, a sense of tightness, or other changes of the skin around the surgical incision or pedestal site. In many cases, these were caused by overly enthusiastic preventive care of the pedestal site by a caretaker or family member, and these resolved after reeducating caregivers. One participant was treated with oral antibiotics for a localized skin infection around a pedestal site and required no further treatment. In more than 17,000 array-days of implant, there were no intracranial or deep tissue (e.g., bone) infections related to the investigational device or any device-related infections requiring hospitalization, intravenous antibiotics, or device removal.

Seven participants had perioperative AEs. In most cases, these were common postoperative problems such as low-grade fever, nausea, or headache. One participant had postoperative hypertension that required treatment with intravenous antihypertensives. A participant with a history of pulmonary embolism who was on chronic anticoagulation developed respiratory distress on postoperative day 3 (before the planned resumption of therapeutic anticoagulation on postoperative day 14). He was diagnosed with recurrent pulmonary embolus and restarted therapeutic anticoagulation with resolution of his symptoms. Given that anticoagulation was held for device placement, this event was classified as related to the study.

In 3 of the 5 participants who had elective array explantation after more than a year (a sixth participant had the pedestal but not the array explanted), array removal was without complications or AEs. In all participants who had elective array removal, it was noted that the arachnoid had grown over the array. For 1 participant who had the BrainGate array explanted at the conclusion of her participation in the trial, surgery revealed that the array was well adhered to the brain and that, as expected, a thin, semitranslucent layer of arachnoid had grown over the array. The arachnoid was incised to partially expose the array, which was then removed. A small piece of tissue secured within the forceps (approximately 5 × 5 × 1 mm intact; measured after dislodgement from the sensor as a triangular piece of tissue 7.0 mm in its longest dimension) was lightly adherent to the electrode side of the array. Histology confirmed that the tissue included meninges and underlying superficial cortex. There was no clinical impact on the participant, who returned to her residence and usual activities on the first postoperative day. In another participant who underwent explantation at the conclusion of trial participation, after removal of the array and all visible portions of reference wires, the surgical investigator noted that the dura was densely adherent to the brain in the area surrounding the array; it was felt unsafe to perform further dissection to retrieve the full length of the 2 15 cm × 0.127-mm diameter reference wires. Postoperative CT imaging demonstrated a short curvilinear hyperdensity within the extra-axial space, suggestive of either a short length of retained reference wire or calcification along the track of the prior location of a reference wire. There were no clinical sequelae.

Two participants, both of whom had a history of traumatic brain injury in addition to SCI, had seizures in the postimplantation period. In one case, the participant was noted intraoperatively to have a small but nonetheless atypical quantity (approximately 2 mL) of subarachnoid hemorrhage around 1 of the 2 implanted arrays. Four days later, he experienced 2 self-limited focal motor seizures, for which he was hospitalized and treated with levetiracetam. He continued levetiracetam as an outpatient, experienced no further seizures, and subsequently participated in the trial for 17 months without any other neurologic AEs. This participant had not received a prophylactic antiseizure medication postoperatively because this practice had not yet been incorporated into the study protocol. Another participant had a single seizure on postoperative day 4; he too was admitted to the hospital, started on levetiracetam, and had no further seizures. He remains enrolled in the clinical trial and has been participating in research sessions for more than 2 years. He had been prescribed a prophylactic antiseizure medication, but 2 doses were missed during the transition from hospital to home. These participants' prior traumatic brain injuries were believed to have increased their risk for seizures.

Before the 2 postoperative seizures described earlier, 1 participant experienced new-onset refractory status epilepticus (NORSE).^11^ This person had quadriplegia and locked-in syndrome due to a brainstem stroke 5 years before enrollment. He had no history of seizures, traumatic brain injury, or other known predisposing comorbidities. After placement of a single array, he had been participating in research sessions for more than 18 months when this SAE occurred. On the morning of the event, the participant's family awoke to a ventilator dyssynchrony alarm and found him unresponsive, with chaotic eye movements. He was transported to Massachusetts General Hospital, where EEG demonstrated status epilepticus. He was treated with phenytoin, levetiracetam, lacosamide, and phenobarbital and intravenous anesthetics (propofol, midazolam, and ketamine) to achieve a burst-suppression pattern on EEG. The pedestal site was clean, and there was no evidence of localized skin infection or irritation. A CT scan of the brain demonstrated no acute structural pathology, and CSF analysis revealed normal levels of protein and glucose, 3–5 white blood cells per microliter, and negative results for viral and bacterial testing. Ultimately, the etiology of his seizures remained unknown, although pneumonia or urinary tract infections were included in the differential diagnosis. Continuous EEG monitoring demonstrated a left temporal lobe origin of his seizures, and the epileptologists involved in his care noted that the cortical location of the BrainGate array (the “hand-knob” of the precentral gyrus of the left frontal lobe) was comparatively less involved in ictal activity. By hospital day 4, he had resolution of all seizure activity. His EEGs evolved after the period of burst-suppression and initially showed generalized slowing with frequent epileptiform discharges; over subsequent days, the background rhythm improved considerably with re-emergence of a posterior dominant rhythm and only rare low-amplitude sharp waves. His antiseizure regimen was slowly tapered down to only lacosamide, and he was discharged to a rehabilitation setting. He resumed participating in research sessions approximately 4 months after this AE and participated in research sessions for another 8 months before concluding his participation in the trial.

While not an AE, we also note that for the participants who had the investigational device (array(s) and/or pedestal) electively explanted at their conclusion of participation in the trial, additional surgical consideration was required to close the well-healed scalp site(s) after removal of the pedestal(s). In participants with a thicker scalp, clean debridement and closure of the healed pedestal site was directly achieved. In a participant with a thinner scalp, additional loosening of the underlying tissue was necessary to close the site, which contributed to postoperative headaches. In addition, and while also not AEs, 2 participants in the first IDE had unexpected abrupt changes (or initial absence) of recording quality. In 1 participant, nonoperative provisional repair of an external component of the percutaneous pedestal was performed 6 months after device placement; the wire bonds had failed at the landing grid assembly within the pedestal, and conductive epoxy was manually placed on each bond site to attempt to restore connections. The initial source of this failure was attributed to the method of sterilization of the devices, which was subsequently changed. Approximately 4.5 months later, the number of useable signals recorded from the array again dropped abruptly, with moderate recovery of signals. There was no clinical change in the participant coinciding with this change in signals nor was this event believed to represent a risk to the health, safety, or welfare of the participant, and it was reported as an unexpected change in device performance. It is unknown whether this loss of signals was due to failure of the previous repair or an unrelated event, such as array dislodgement/displacement from coughing (which the patient had reported) or other participant-specific factors. After the initial device failure and discussion with the device manufacturer, modifications were made to the sterilization procedures used before delivery to investigators. In a second participant during this early period, caregivers inadvertently applied a sudden axial force to the participant's head and pedestal while placing the participant back into bed (by too forcefully contacting the headrest of the bed with the participant's head). There was a substantial return of recording quality within a month, though with considerably more day-to-day variability than previously seen. Overall, this study provides Class IV evidence that the neurosurgically placed BrainGate Neural Interface system is associated with a low rate of SAEs, defined as those requiring device explantation, resulting in death, or resulting in permanently increased disability during the 1-year postimplant period.

## Discussion

The investigational BrainGate Neural Interface system has an encouraging safety record, with more than 17,000 person-array implant-days of data. All 12 participants who were enrolled for more than 1 year met the primary safety endpoint of not having the device removed for safety reasons during the 1-year postimplant evaluation. Although 6 SAEs were classified as possible, probably, or definitely device-related or procedure-related, there were no device-related SAEs that resulted in death or permanently increased disability during the 1-year postimplant evaluation period (Two participants died of complications from ALS before the 1-year evaluation). Device safety has also remained reassuring over the 2 to 5+ years that 8 participants have been enrolled.

The most common AE (Table 4) was local skin reaction and/or sensitivity around the pedestal, in most cases related to expected postsurgical changes or excessive cleaning with chlorhexadine solution as part of the infection control protocol. The most notable adverse safety events were postoperative seizures that occurred 4 days postdevice implantation in 2 participants, both of whom had a history of traumatic brain injury and had not received consistent postoperative seizure prophylaxis. Neither participant had any further seizures nor did these events affect their ability to continue to participate in the clinical trial or in their usual activities of daily living. Although preclinical data did not suggest the need for seizure prophylaxis, the trial protocol now includes 1 week of postoperative prophylactic antiseizure medication (a common practice for supratentorial craniotomies). In a participant who had a BrainGate device for more than 5 years, a small amount of tissue was adherent to the array during explantation; for 4 other participants, the array was removed without any adherent tissue. A recently published study demonstrated comparable findings in 6 Utah arrays explanted from 2 other human research participants,^12^ which may be an important consideration as this technology moves toward wider use.

**Table 4:**
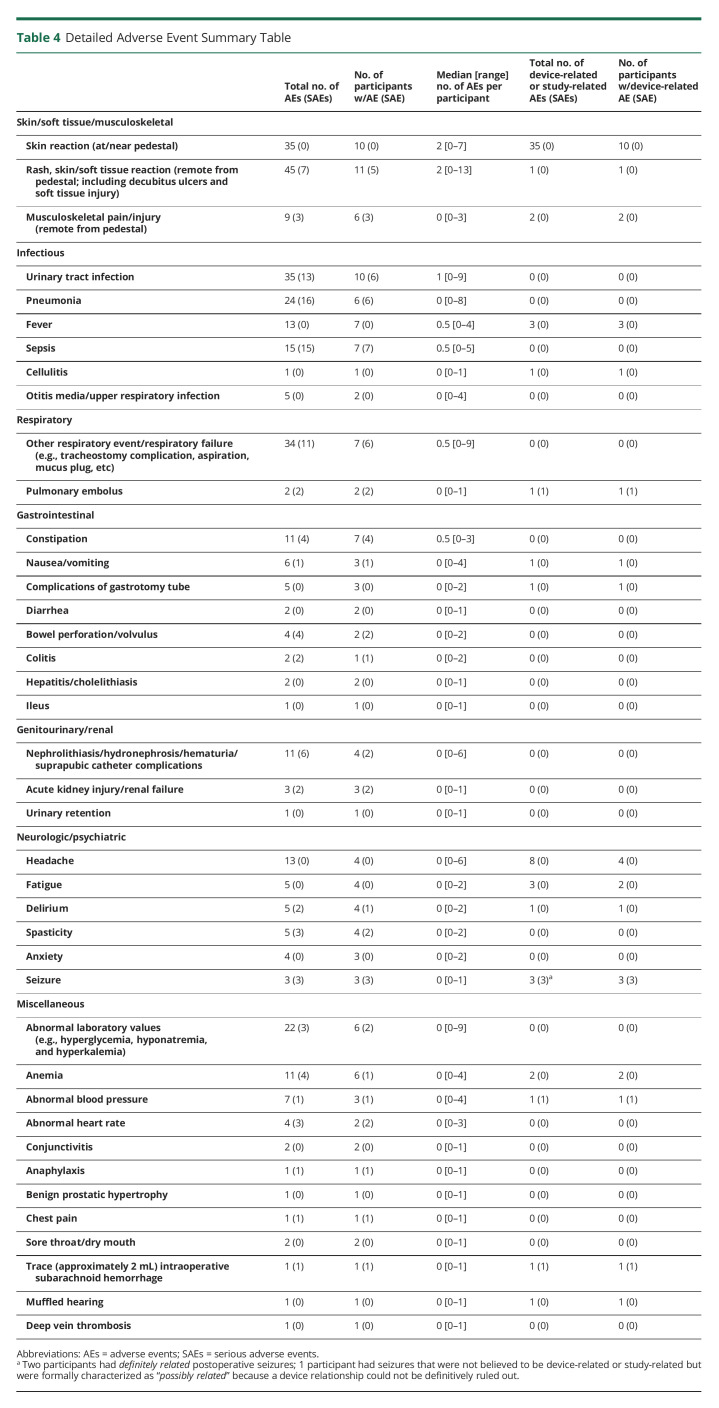
Detailed Adverse Event Summary Table

The participant who experienced NORSE illustrates how medical device research can raise dilemmas at the interface of research ethics and medical ethics. Similar to many trials of novel medical devices, the ethical premise under which participants are recruited and provide informed consent to this study is that there *is risk* to participation and *no benefit* to the individual; the only benefit is that gained by society through the development of a system that could improve communication and/or mobility for people with paralysis. In this context, the NORSE SAE raised important questions that required immediate decisions. As with any clinical trial, an SAE of uncertain relationship to study procedures required rapid and comprehensive evaluation. The decision whether to remove the array was explicitly made on clinical grounds alone, and on this basis, the recommendation was to not remove the array because the neurosurgical intervention would be without a clear clinical indication and could increase the risk for additional seizures and other complications. However, during extensive discussions with the participant's family, they conveyed that he had repeatedly expressed enjoyment and altruistic fulfillment by contributing to scientific advances that could benefit people with paralysis and would be saddened to learn that he could no longer participate in the trial because the investigational device had been removed. While his family's impression of the best patient/participant–centered approach was consistent with the clinical impression for managing this SAE, the investigational team realized that the original “no benefit” premise did not fully capture the factors involved in deciding whether to remove the device. The presence of personal intangible benefits such as altruistic fulfillment might be considered in the design, review, and conduct of trials that seem to be without benefit to individuals considering enrollment.

This work has some limitations warranting discussion. The most significant, from a clinical trials standpoint, is the lack of a control arm; for myriad ethical and logistical reasons, an experimental control (e.g., surgery without device use) would not be feasible. We are nonetheless reassured by our findings that the percutaneous BrainGate system has a safety profile, to date, in a population of people with tetraplegia, which is comparable with fully implanted deep brain stimulation (DBS) devices approved for the treatment of certain movement disorders and the responsive neurostimulation (RNS) device approved for the treatment of partial-onset seizures refractory to 2 or more antiepileptic medications. Large studies with more than 100 patients undergoing DBS implantation have reported infection rates between 3% and 5% of patients,^6,13-15^ of which up to half have required hardware removal.^15^ Other complications included subcortical hemorrhage, subdural hematoma, venous infarction, and seizure; these were observed in 1%–5% of patients in case series.^16,17^ A meta-analysis of 1,714 patients who underwent DBS for essential tremor reported an infection rate of 3.4%, hemorrhage rate of 2.4%, and seizure rate of 2.3%.^18^ Device-specific complications, such as lead migration, misplaced leads, and implantable pulse generator failure have occurred at rates of approximately 3%–5%.^19^ In the long-term trial of the NeuroPace RNS, a chronically implanted neurostimulator used in the treatment of medication refractory epilepsy, the only device-related SAE observed in >5% of the 230 enrolled participants was implantation site infection (in 12.1%),^20^ with no instances of meningitis or parenchymal brain infection (although bone flap osteomyelitis was observed).^21^ Some patients also required operative repair of damaged leads.^22^ Seven patients (2.7%) had hemorrhage, 4 of which were associated with implantation surgery and had no neurologic sequaelae.^20^

Our clinical research experience with a percutaneously connected intracortical neural recording system has included only a single device-related skin infection and no bone or CNS infections. We ascribe this to a thorough preoperative skin cleansing protocol (consisting of 3 days of a daily pre-operative full body wash with chlorhexidine soap); careful intraoperative attention to sterile procedure; a rigorous caregiver protocol for maintaining an aseptic environment around the percutaneous pedestals; and chlorhexidine-impregnated patches (BioPatch) around the pedestal site (Table 5). However, we recognize that our study is underpowered to detect rare events such as intracranial infection. Given the low incidence in the general population (1.38 cases per 100,000 population per year in 2006–2007),^23^ our current study, with 33.43 person-years of observation, has a power of less than 1% to detect a doubling in the rate of intracranial infection at a 0.05 level of significance. Had we observed intracranial infections, we could query for a statistical deviation from the background rate, if present.

**Table 5:**
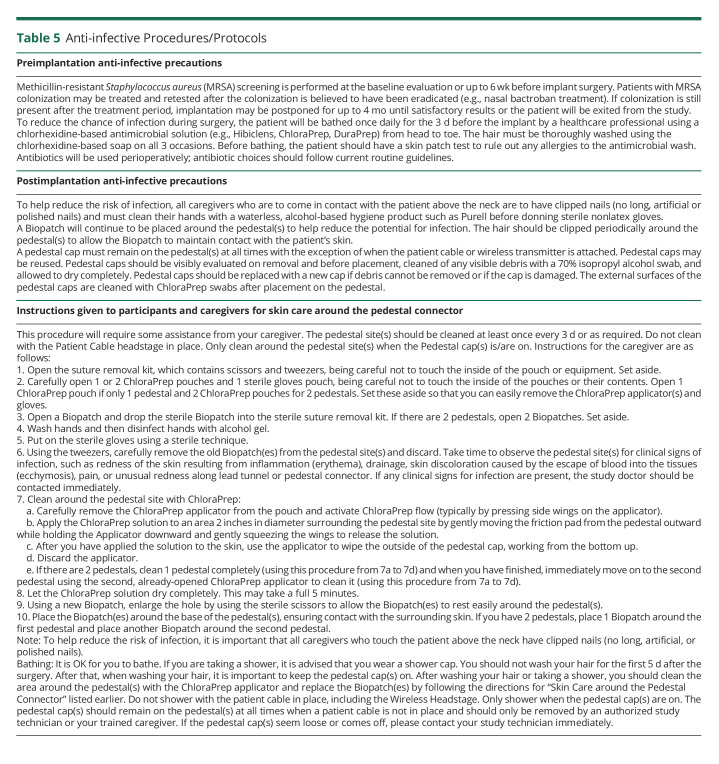
Anti-infective Procedures/Protocols

A second limitation in comparing the safety profile of this device with other chronically implanted neurotechnologies is that the people enrolled in the BrainGate trial have different neurologic conditions than the populations enrolled in other neurotechnology trials. People with quadriparesis may have greater contact with caregivers attending to health-related needs than those with epilepsy or Parkinson disease; consequently, comparisons with devices intended for ambulatory populations are imperfect. In addition, while our participants consented to the risk of worsening paralysis because of array placement on motor cortex (which has not been observed), given the significant baseline weakness required to enroll, subtle changes in motor function may occur below the threshold of clinical perception. With that said, the ongoing BrainGate trial and subsequent studies using similar research protocols and the Blackrock NeuroPort array were informed by numerous animal studies of safety, histology, and chronic device function^24-27^ and with the understanding that other research studies for participants with paralysis who could benefit from future intracortical BCI devices would continue to inform the field.^9,28-30^ In addition to our 14 participants, as of April 2022, there have been approximately 21 other human research participants who have participated in chronic intracortical BCI trials at academic centers in the United States,^28,29,31-33^ China,^34^ and Germany.^35^ Chronically implanted electrocorticography grids have been placed for similar research in the United States and The Netherlands,^31,36^ and 4 investigational Stentrode devices have been placed in Australia.^37,38^ Several neurotechnology companies are also preparing clinical trials of implanted BCI systems, reinforcing the growing interest in developing and deploying a clinically useful implanted BCI.

While safety is the *sine qua non* of any successful medical technology, quantifiable metrics of efficacy are important to establish clinical utility and to help individuals to evaluate the technology within their personal value system of risks and benefits. Our current feasibility study is being conducted partly to inform the development of metrics upon which efficacy could be evaluated in a subsequent pivotal trial. Prior publications from our group and others have demonstrated the potential for intracortical BCIs to improve the quality of life for people with paralysis. With highly accurate 2-dimensional cursor control that can be calibrated within minutes of initial use,^39^ BrainGate participants have controlled a point-and-click interface to communicate at 30–40 correct characters per minute (CPM).^40,41^ Using handwriting decoding techniques, a communication rate of 90 CPM has been demonstrated,^9^ approaching the range of an able-bodied individual typing on a smartphone keyboard.^42^ In addition to developing methods for improved communication, the investigational BrainGate system and related intracortical BCIs have been used to control assistive robotics^7,28,43^ and a participant's own arm and hand through functional electrical stimulation.^33,44^ These demonstrations suggest the potential to provide a meaningful improvement in quality of life.

While an exhaustive review of the potential benefits of the iBCI approach is beyond the scope of this article, the “risk/benefit” analysis—particularly as considered by an individual with paralysis contemplating the use of different assistive technologies—will be central to the future success of these devices. For example, some participants have entered the study frustrated, for a host of reasons, by the utility of their eye-gaze systems. Despite relative preservation of eye movements in the earlier stages of ALS, subtle changes in motor control, slowness of saccades, fatigue in supporting musculature, and progressive ophthalmoparesis can all affect performance with these systems.^45^ Limited data suggest communication rates of 10–20 CPM in people with ALS; in a limited head-to-head study, participants communicated faster using a manual letter board than a computerized eye-gaze system.^46,47^

Furthermore, for a person with severe speech and motor impairments, the ability to use an assistive technology while visually engaging with their dialog partner—which itself has communication value—is also important. Consequently, iBCIs provide not only theoretical advantages over eye-gaze systems but also over other BCIs (implanted or EEG based) that encumber eye movements specifically to navigate communication interfaces. Given iBCIs' early demonstrations of the ability to support multidimensional, dexterous reach-and-grasp movements, to restore sensation (with some further improvement in, for example, assistive robot control),^29^ the broad potential for iBCIs is clear. Despite the flexibility and range of effector control that iBCIs may provide in the future, the current percutaneous component and substantial external hardware will remain suboptimal features when compared with fully implanted or nonsurgery-requiring approaches. Both our group and others continue to work on components and systems that would permit iBCIs to become fully implanted, available to users around-the-clock, and incorporate a suite of design characteristics previously proposed.^48^

Overall, we are reassured by our findings over the past 17 years that the investigational BrainGate Neural Interface System is being deployed safely. The development of a fully implantable system, which would reduce the infection concern presented by a percutaneous component and provide more acceptable cosmesis for many users, remains an important goal of our group and others. Moreover, this trial has been conducted during and continues to benefit from a revolution in machine learning/artificial intelligence and microprocessor technology. Advances in these fields have contributed to the ongoing improvement in performance that we have observed, and we anticipate that these technical gains will only accelerate in the coming years. Of importance, these same technological advances have led to improvements in consumer assistive technology available to people with paralysis. Voice control platforms such as Siri and Alexa may allow a person with quadriplegia and intact speech naturalistic control over their consumer electronics and home environment, and head-mounted virtual reality/augmented reality technology may provide additional channels of communication for people who have lost the ability to speak. However, no one technological solution will work best for everyone. Indeed, many able-bodied individuals prefer to type or text rather than use voice recognition to maintain the privacy of their communication from the people in their immediate surroundings, and much of the drive for iBCIs is not only to restore communication but also to restore mobility through intuitively controlled functional electrical stimulation or soft/wearable robotics. Intracortical BCIs also are being developed as key components of closed-loop neuromodulation therapies that may improve the management of disorders of mood or cognition.^49,50^ Ultimately, the extraordinary potential utility of implanted BCIs will reflect the personal risk/benefit assessment of the people for whom these devices are being developed.

## Glossary

AEs: adverse events
BCIs: brain-computer interfaces
CPM: characters per minute
DBS: deep brain stimulation
IDE: Investigational Device Exemption
NORSE: new-onset refractory status epilepticus
RNS: responsive neurostimulation
SAEs: serious adverse events
SCI: spinal cord injury
UADEs: unanticipated adverse device effects

## References

[1] Hochberg LR, Cudkowicz ME. Locked in, but not out? Neurology. 2014;82(21):1852-1853. doi: 10.1212/wnl.0000000000000460. 24789868

[2] Lee B, Liu CY, Apuzzo MLJ. A primer on brain-machine interfaces, concepts, and technology: a key element in the future of functional neurorestoration. World Neurosurg. 2013;79:457-471. 23333985 10.1016/j.wneu.2013.01.078

[3] Martini ML, Oermann EK, Opie NL, Panov F, Oxley T, Yaeger K. Sensor modalities for brain-computer interface technology: a comprehensive literature review. Neurosurgery. 2020;86:E108–E117. 31361011 10.1093/neuros/nyz286

[4] Brandman DM, Cash SS, Hochberg LR. Review: human intracortical recording and neural decoding for brain-computer interfaces. IEEE Trans Neural Syst Rehabil Eng. 2017:1687-1696. 28278476 10.1109/TNSRE.2017.2677443 PMC5815832

[5] Soldozy S, Young S, Kumar JS, et al. A systematic review of endovascular stent-electrode arrays, a minimally invasive approach to brain-machine interfaces. Neurosurg Focus. 2020;49(1):E3. doi: 10.3171/2020.4.focus20186. 32610291

[6] Bullard AJ, Hutchison BC, Lee J, Chestek CA, Patil PG. Estimating risk for future intracranial, fully implanted, modular neuroprosthetic systems: a systematic review of hardware complications in clinical deep brain stimulation and experimental human intracortical arrays. Neuromodulation. 2020;23:411-426. 31747103 10.1111/ner.13069

[7] Hochberg LR, Serruya MD, Friehs GM, et al. Neuronal ensemble control of prosthetic devices by a human with tetraplegia. Nature. 2006;442(7099):164-171. doi: 10.1038/nature04970. 16838014

[8] Simeral JD, Kim S-P, Black MJ, Donoghue JP, Hochberg LR. Neural control of cursor trajectory and click by a human with tetraplegia 1000 days after implant of an intracortical microelectrode array. J Neural Eng. 2011;8(2):025027. ii: 10.1088/1741-2560/8/2/025027. 21436513 PMC3715131

[9] Willett FR, Avansino DT, Hochberg LR, Henderson JM, Shenoy KV. High-performance brain-to-text communication via handwriting. Nat Res. 2021;593(7858):249-254. doi: 10.1038/s41586-021-03506-2. PMC8163299 33981047

[10] Simeral JD, Hosman T, Saab J, et al. Home use of a percutaneous wireless intracortical brain-computer interface by individuals with tetraplegia. IEEE Trans Biomed Eng IEEE Comput Soc. 2021;68(7):2313-2325. doi: 10.1109/tbme.2021.3069119. PMC8218873 33784612

[11] Costello DJ, Kilbride RD, Cole AJ. Cryptogenic new onset refractory status epilepticus (NORSE) in adults-infectious or not? J Neurol Sci. 2009;277(1-2):26-31. doi: 10.1016/j.jns.2008.10.007. 19013586

[12] Woeppel K, Hughes C, Herrera AJ, et al. Explant analysis of UT electrode arrays implanted in human cortex for brain-computer-interfaces. Front Bioeng Biotechnol. 2021;9:759711-759715. doi: 10.3389/fbioe.2021.759711. 34950640 PMC8688945

[13] Son BC, Shon YM, Choi JG, et al. Clinical outcome of patients with deep brain stimulation of the centromedian thalamic nucleus for refractory epilepsy and location of the active contacts. Stereotact Funct Neurosurg. 2016;94(3):187-197. doi: 10.1159/000446611. 27434073

[14] Lyons KE, Wilkinson SB, Overman J, Pahwa R. Surgical and hardware complications of subthalamic stimulation: a series of 160 procedures. Neurology. 2004;63(4):612-616. doi: 10.1212/01.wnl.0000134650.91974.1a. 15326230

[15] Vergani F, Landi A, Pirillo D, Cilia R, Antonini A, Sganzerla EP. Surgical, medical, and hardware adverse events in a series of 141 patients undergoing subthalamic deep brain stimulation for Parkinson disease. World Neurosurg. 2010;73(4):338-344. doi: 10.1016/j.wneu.2010.01.017. 20849789

[16] Umemura A, Jaggi JL, Hurtig HI, et al. Deep brain stimulation for movement disorders: morbidity and mortality in 109 patients. J Neurosurg. 2003;98(4):779-784. doi: 10.3171/jns.2003.98.4.0779. 12691402

[17] Seijo FJ, Alvarez-Vega MA, Gutierrez JC, Fdez-Glez F, Lozano B. Complications in subthalamic nucleus stimulation surgery for treatment of Parkinson's disease. Review of 272 procedures. Acta Neurochir (Wien). 2007;149(9):867-876. doi: 10.1007/s00701-007-1267-1. 17690838

[18] Lu G, Luo L, Liu M, et al. Outcomes and adverse effects of deep brain stimulation on the ventral intermediate nucleus in patients with essential tremor. Neural Plast. 2020;2020:2486065. 32802034 10.1155/2020/2486065 PMC7416257

[19] Doshi PK. Long-term surgical and hardware-related complications of deep brain stimulation. Stereotact Funct Neurosurg. 2011;89(2):89-95. doi: 10.1159/000323372. 21293168

[20] Nair DR, Laxer KD, Weber PB, et al. Nine-year prospective efficacy and safety of brain-responsive neurostimulation for focal epilepsy. Neurology. 2020;95(9):e1244-e1256. doi: 10.1212/wnl.0000000000010154. 32690786 PMC7538230

[21] Degenhart AD, Eles J, Dum R, et al. Histological evaluation of a chronically-implanted electrocorticographic electrode grid in a non-human primate. J Neural Eng. 2016;13:046019. 27351722 10.1088/1741-2560/13/4/046019 PMC4993459

[22] Lee B, Zubair MN, Marquez YD, et al. A single-center experience with the NeuroPace RNS system: a review of techniques and potential problems. World Neurosurg. 2015;84:719-726. 25940211 10.1016/j.wneu.2015.04.050

[23] Thigpen MC, Whitney CG, Messonnier NE, et al. Bacterial meningitis in the United States, 1998-2007. N Engl J Med. 2011;364(21):2016-2025. doi: 10.1056/nejmoa1005384. 21612470

[24] Suner S, Fellows MR, Vargas-Irwin C, Nakata GK, Donoghue JP. Reliability of signals from a chronically implanted, silicon-based electrode array in non-human primate primary motor cortex. IEEE Trans Neural Syst Rehabil. 2005;13(4):524-541. doi: 10.1109/tnsre.2005.857687. 16425835

[25] Rousche PJ, Normann RA. Chronic intracortical microstimulation (ICMS) of cat sensory cortex using the Utah intracortical electrode array. IEEE Trans Rehabil Eng IEEE. 1999;7(1):56-68. doi: 10.1109/86.750552. 10188608

[26] Rousche PJ, Normann RA. Chronic recording capability of the Utah intracortical electrode array in cat sensory cortex. J Neurosci Methods. 1998;82:1-15. 10223510 10.1016/s0165-0270(98)00031-4

[27] Barrese JC, Rao N, Paroo K, et al. Failure mode analysis of silicon-based intracortical microelectrode arrays in non-human primates. J Neural Eng. 2013;10(6):066014. doi: 10.1088/1741-2560/10/6/066014. 24216311 PMC4868924

[28] Collinger JL, Wodlinger B, Downey JE, et al. High-performance neuroprosthetic control by an individual with tetraplegia. Lancet. 2013;381(9866):557-564. doi: 10.1016/s0140-6736(12)61816-9. 23253623 PMC3641862

[29] Flesher SN, Downey JE, Weiss JM, et al. A brain-computer interface that evokes tactile sensations improves robotic arm control. Science. 2021;372(6544):831-836. doi: 10.1126/science.abd0380. 34016775 PMC8715714

[30] Bouton CE, Shaikhouni A, Annetta NV, et al. Restoring cortical control of functional movement in a human with quadriplegia. Nature. 2016;533(7602):247-250. doi: 10.1038/nature17435. 27074513

[31] Moses DA, Metzger SL, Liu JR, et al. Neuroprosthesis for decoding speech in a paralyzed person with anarthria. N Engl J Med. 2021;385(3):217-227. doi: 10.1056/nejmoa2027540. 34260835 PMC8972947

[32] Cajigas I, Davis KC, Meschede-Krasa B, et al. Implantable brain–computer interface for neuroprosthetic-enabled volitional hand grasp restoration in spinal cord injury. Brain Commun. 2021;3(4):fcab248. doi: 10.1093/braincomms/fcab248. 34870202 PMC8637800

[33] Colachis SC, Bockbrader MA, Zhang M, et al. Dexterous control of seven functional hand movements using cortically-controlled transcutaneous muscle stimulation in a person with tetraplegia. Front Neurosci. 2018;12:208-214. doi: 10.3389/fnins.2018.00208. 29670506 PMC5893794

[34] Jiang H, Wang R, Zheng Z, et al. Short report: surgery for implantable brain-computer interface assisted by robotic navigation system. Acta Neurochir (Wien). 2022;164(9):2299-2302. doi: 10.1007/s00701-022-05235-5. 35604492

[35] Chaudhary U, Vlachos I, Zimmermann JB, et al. Spelling interface using intracortical signals in a completely locked-in patient enabled via auditory neurofeedback training. Nat Commun. 2022;13:1236-1239. doi: 10.1038/s41467-022-28859-8. 35318316 PMC8941070

[36] Vansteensel MJ, Pels EGM, Bleichner MG, et al. Fully implanted brain–computer interface in a locked-in patient with ALS. N Engl J Med. 2016;375(21):2060-2066. doi: 10.1056/nejmoa1608085. 27959736 PMC5326682

[37] Oxley TJ, Yoo PE, Rind GS, et al. Motor neuroprosthesis implanted with neurointerventional surgery improves capacity for activities of daily living tasks in severe paralysis: first in-human experience. J Neurointerv Surg. 2021;13(2):102-108. doi: 10.1136/neurintsurg-2020-016862. 33115813 PMC7848062

[38] Campbell B, Lee CMS, Yoo P, et al. Long-term safety of a fully implanted endovascular brain-computer interface for severe paralysis: results of SWITCH, a first-in-human study. Paper presented at: 74th American Academy of Neurology Annual Meeting; April 2–7, 2022; Seattle, WA.

[39] Brandman DM, Hosman T, Saab J, et al. Rapid calibration of an intracortical brain-computer interface for people with tetraplegia. J Neural Eng. 2018;15(2):026007-026026. doi: 10.1088/1741-2552/aa9ee7. 29363625 PMC5823702

[40] Pandarinath C, Nuyujukian P, Blabe CH, et al. High performance communication by people with paralysis using an intracortical brain-computer interface. Elife. 2017;6:185544-e18627. doi: 10.7554/elife.18554. PMC5319839 28220753

[41] Bacher D, Jarosiewicz B, Masse NY, et al. Neural point-and-click communication by a person with incomplete locked-in syndrome. Neurorehabil Neural Repair. 2015;29(5):462-471. doi: 10.1177/1545968314554624. 25385765 PMC4426256

[42] Palin K, Feit AM, Kim S, Kristensson PO, Oulasvirta A. How do people type on mobile devices? Observations from a study with 37, 000 volunteers. Paper presented at: 21st International Conference on Human-Computer Interaction with Mobile Devices and Services, MobileHCI 2019; October 1–4, 2019; Taipei, Taiwan. 10.1145/3338286.3340120

[43] Hochberg LR, Bacher D, Jarosiewicz B, et al. Reach and grasp by people with tetraplegia using a neurally controlled robotic arm. Nature. 2012;485:372-375. 22596161 10.1038/nature11076 PMC3640850

[44] Ajiboye AB, Willett FR, Young DR, et al. Restoration of reaching and grasping movements through brain-controlled muscle stimulation in a person with tetraplegia: a proof-of-concept demonstration. Lancet. 2017;389(10081):1821-1830. doi: 10.1016/s0140-6736(17)30601-3. 28363483 PMC5516547

[45] Linse K, Aust E, Joos M, Hermann A. Communication matters—pitfalls and promise of hightech communication devices in palliative care of severely physically disabled patients with amyotrophic lateral sclerosis. Front Neurol. 2018;9:603. doi: 10.3389/fneur.2018.00603. 30100896 PMC6072854

[46] Pannasch S, Helmert JR, Malischke S, Storch A, Velichkovsky BM. Eye typing in application: a comparison of two systems with ALS patients. J Eye Mov Res. 2008;2(4):1-8. doi: 10.16910/jemr.2.4.6.

[47] Harris D, Goren M. The ERICA eye gaze system versus manual letter board to aid communication in ALS/MND. Br J Neurosci Nurs. 2009;5:227-230. doi: 10.12968/bjnn.2009.5.5.42128.

[48] Hochberg LR, Anderson K. BCI users and their needs. In: Wolpaw JR, Wolpaw E, eds. Brain-Computer Interfaces: Principles and Practice Hardcover. Oxford University Press; 2012.

[49] Scangos KW, Khambhati AN, Daly PM, et al. Closed-loop neuromodulation in an individual with treatment-resistant depression. Nat Med. 2021;27(10):1696-1700. doi: 10.1038/s41591-021-01480-w. 34608328 PMC11219029

[50] Drew L. Wiring up the brain to beat depression. Nature. 2022;608(7924):S46-S47. doi: 10.1038/d41586-022-02209-6. 36002493

